# Conformational Motion of the ABC Transporter MsbA Induced by ATP Hydrolysis

**DOI:** 10.1371/journal.pbio.0050271

**Published:** 2007-10-09

**Authors:** Peter P Borbat, Kavitha Surendhran, Marco Bortolus, Ping Zou, Jack H Freed, Hassane S Mchaourab

**Affiliations:** 1 Department of Chemistry and Chemical Biology, Baker Laboratory, Cornell University, Ithaca, New York, United States of America; 2 Department of Molecular Physiology and Biophysics, Vanderbilt University Medical Center, Nashville, Tennessee, United States of America; Brandeis University, United States of America

## Abstract

We measured the amplitude of conformational motion in the ATP-binding cassette (ABC) transporter MsbA upon lipopolysaccharide (LPS) binding and following ATP turnover by pulse double electron-electron resonance and fluorescence homotransfer. The distance constraints from both methods reveal large-scale movement of opposite signs in the periplasmic and cytoplasmic part of the transporter upon ATP hydrolysis. LPS induces distinct structural changes that are inhibited by trapping of the transporter in an ATP post-hydrolysis intermediate. The formation of this intermediate involves a 33-Å distance change between the two ABCs, which is consistent with a dimerization-dissociation cycle during transport that leads to their substantial separation in the absence of nucleotides. Our results suggest that ATP-powered transport entails LPS sequestering into the open cytoplasmic chamber prior to its translocation by alternating access of the chamber, made possible by 10–20-Å conformational changes.

## Author Summary

Clinical multidrug resistance in the treatment of bacterial and fungal infections and cancer chemotherapy can result from the expression of pumps that extrude toxic molecules from the cell. A subclass of these pumps—ATP-binding cassette (ABC) transporters—use energy from ATP to remove a wide range of molecules. MsbA is a conserved ABC transporter from Gram-negative bacteria with sequence similarity to human multi-drug ABC transporters. MsbA flips the building block of the outer membrane, lipid A, across the inner membrane. The input of ATP energy occurs in two dedicated nucleotide-binding domains (NBDs), whose configuration in intact transporters is controversial. We determined the amplitude of MsbA conformational motion that couples energy expenditure to substrate movement across the membrane. Using molecular probes introduced into the protein sequence, we found that ATP hydrolysis fuels a relative motion of the NBDs close to 30 Å. The movement of the NBDs is coupled to reorientation of the chamber, which binds the lipid substrate from cytoplasmic-facing to extracellular-facing through large amplitude motion on either side of the transporters. In addition to revealing the structural mechanics of transport, these results challenge current models deduced from studies of substrate-specific ABC importers that envisions the two NBDs in contact throughout the ATP hydrolysis cycle.

## Abstract 3

Analysis of the conformational changes that occur in a conserved ATP-binding cassette (ABC) transporter challenges current models of this clinically important class of molecules.

## Introduction

ATP-binding cassette (ABC) transporters harness the free energy of ATP hydrolysis to power the thermodynamically unfavorable trafficking of a wide spectrum of substrates in and out of the cell [1–3]. Cooperative ATP binding and hydrolysis occur in a molecular motor composed of two ATP-binding and hydrolysis cassettes (ABCs), also referred to as nucleotide-binding domains (NBDs) [4]. ATP binds at the interface of an NBD dimer sandwiched between the Walker A motif of one subunit and the signature motif of the symmetry-related subunit [5–7]. Two transmembrane domains encode the determinants of substrate binding and provide a passageway across the bilayer.

In Gram-negative bacteria, the transport of lipid A from its site of synthesis across the inner membrane is critically dependent on the expression of the ABC transporter MsbA. Loss of MsbA activity inhibits growth and is associated with the accumulation of lipid A in the cytoplasmic leaflet of the inner membrane [8–10]. MsbA has sequence similarity to a subclass of ABC transporters that is linked to the development of multidrug resistance in microorganisms and cancer through the extrusion of structurally dissimilar molecules [11,12]. Polyspecifity appears to be a common property of ABC efflux systems, whereas importers are substrate specific, often requiring a dedicated high-affinity binding protein for substrate delivery [1].

The molecular organization of the four domains of ABC transporters was gleaned from crystal structures of a number of importers as well as the bacterial multidrug efflux system Sav1866 [13–15]. The structures confirm the canonical interface observed in isolated NBD dimers [4,16], identify structural elements in the cytoplasmic side that mediate communication between the NBDs and the transmembrane domains, and define the likely pathway of substrate transport and putative gates that control substrate access. Initial structures of MsbA proved incompatible with biochemical and structural data and were subsequently retracted owing to an analysis error [17–20]. A large body of biochemical studies including cryo–electron microscopy (EM) analysis [21], cross-linking studies of P-glycoprotein (P-gp) [22–25], and kinetic and thermodynamic analysis of P-gp and LmrA substrate transport cycles [2,26] have delineated many aspects of the transport mechanism. They collectively demonstrate that the energy input of ATP binding and/or hydrolysis is transduced to the mechanical work of an inward-outward–facing cycle of the substrate binding sites [27].

Structural studies of MsbA by site-directed spin labeling [28] and electron spin resonance (ESR) spectroscopy suggested that in liposomes, MsbA undergoes substantial conformational changes upon ATP binding and hydrolysis [29]. Reporting on the accessibilities and relative proximities of three transmembrane helices—2, 5, and 6—and adjacent regions of the intracellular domain and periplasmic loops, the spin labels revealed the presence of an asymmetric, water-exposed chamber that is open to the cytoplasm in the absence of nucleotides. ATP binding or hydrolysis occludes the chamber to the cytoplasm and increases hydration in the periplasmic side along an alternating access model [29]. Accessibility changes are accompanied by opposite proximity changes on the cytoplasmic and periplasmic sides of the transporter, although the amplitude of these movements was not determined. Conformational changes induced by either ATP binding or by the formation of a ADP/Vanadate (Vi) post-hydrolysis intermediate are of similar sign and magnitude, suggesting that the ATP binding provides the power stroke for transport as previously reported for P-gp [21]. The ESR constraints indicate that apo-MsbA samples conformations that depart from the reported crystal structures in helix topology and the extent of opening on the extracellular side [29]. In particular, these experiments indicated that helix 6 is shielded from direct exposure to the bilayer and is likely packed at the dimer interface as concluded from cross-linking studies of P-gp and subsequently confirmed by the Sav1866 structure [13,30].

Recent crystal structures have captured importers in inward-facing and outward-facing conformations and presented a model of the amplitude and extent of the underlying structural changes [15]. A central theme of this model is the limited rearrangement of the NBD dimer interface upon ATP binding and hydrolysis. Whether this model applies for efflux ABC transporters is yet to be determined. The Sav1866 structure [13] corresponds to a post-hydrolysis intermediate; thus it does not define the amplitude of the movement associated with chamber reorientation nor does it address the critical question of whether the two NBD domains remain closely packed during the ATPase cycle. In addition, the crystal structures were all determined in detergent micelles and thus may be influenced by the absence of lipids in addition to conformational selectivity imposed by crystal lattice forces [15,31].

To further map the conformational changes in the transport cycle of MsbA, we used pulse dipolar ESR spectroscopy [32–36] and fluorescence homotransfer [37–39] to obtain a set of critical distance constraints that monitor the relative separation of the transmembrane domains, the dimer interface, and the packing of the NBDs in detergent micelles and in the native-like environment of lipid bilayers. The distances were determined following the addition of a putative substrate, LPS, and in the high-energy post ATP-hydrolysis intermediate in the presence and absence of LPS. The change in the distance constraints provides evidence of LPS interaction with the transporter and establishes the sign and amplitude of the conformational changes on the cytoplasmic and periplamic sides following ATP hydrolysis.

## Results

### Methodology

Spin and fluorescence labels were attached to single cysteines in each MsbA monomer, resulting in the introduction of two symmetry-related probes in the functional dimeric unit. Distances between the probes were determined by ESR and fluorescence spectroscopies in detergent micelles. For a selected set, distances between spin labels were also measured in liposomes to establish the correspondence with conformational changes in detergent micelles. The mutants selected for this study form dimers as demonstrated by their retention times on size-exclusion chromatography. Spin-labeled mutants were shown previously to turn over ATP with rates ranging from 20%–100% of that of the wild type (WT) and at least 10-fold higher than the Vi-inhibited WT [29].

Three nucleotide-bound intermediates of MsbA can be stably populated: ATP-bound, ADP-bound, and ADP/Vi-inhibited. The latter is a high-energy post-hydrolysis intermediate often referred to as the transition state of ATP hydrolysis [40]. Previous site-directed spin labeling data indicated that ATP binding and hydrolysis are associated with similar overall conformational changes in MsbA [29]. Therefore, here we focus on the ADP/Vi intermediate.

### Amplitude of Conformational Changes Induced by ATP Hydrolysis

The sites for distance measurements were selected in regions that report changes in the local environment of spin labels [29] following the formation of the ADP/Vi intermediate. These include the intracellular or cytoplasmic sides (IL in the notation of Dawson and Locher [13]) of helices 2, 5, and 6, where an overall reduction in accessibility to nickel-ethylenediaminediacetic acid (NiEDDA) was observed, and the periplasmic loop 2 (ECL1), where increased water accessibility accompanies ATP binding and hydrolysis.

**Figure 1:**
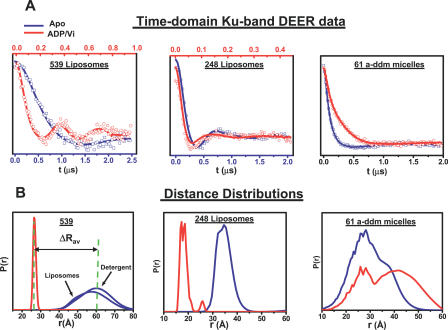
Representative Distance Changes in MsbA Induced by ATP Hydrolysis (A) Ku-band (17.3 GHz) DEER signals at representative sites illustrating the change in the signal induced by the transition from the apo state to the ADP/Vi intermediate. (B) In this transition, distance distributions *P*(*r*) for sites 539 and 248 become narrow, with average distances showing large negative shifts; whereas for site 61, the shift is large and positive. Time-domain DEER signals and details of distance analysis are further illustrated in Figures S1–S5 supplementary material. The broad distance distribution at site 61 indicates a highly flexible loop. The distance change at this site in liposomes (even though it is very similar to that in detergent) may indicate a shift in the relative population in a bimodal distribution (Figure S1) although further work would be required to substantiate this result. In the samples of ADP/Vi intermediates, a small fraction of MsbA in the apo state is also present; and as a result, a low-intensity component in the *P*(*r*), corresponding to this state, is visible in this case and is larger in lipid. (These satellites are suppressed for sites 248 and 539 in (B).) For site 61, the lines overlap and are displayed as is, but they are shown separated in the supplement. a-ddm: alpha dodecyl maltoside.

Double electron-electron resonance (DEER) data in liposomes and detergent micelles were obtained in the apo state and following ATP hydrolysis and Vi trapping to form a high-energy ADP/Vi intermediate. Figure 1A shows a representative set of data, and Figure 1B shows distance distributions calculated as previously described [41]. The length of data records displayed in Figure 1A was selected to optimize the trade-off between the signal-to-noise ratio and attainable range and resolution of distances [34,42]. We observed distinct dipolar oscillations in the DEER signal for all sites (except 61) in the post-hydrolysis intermediate. They were less distinct in the apo state, and aperiodic decays for the loop site 61 are typical of a wide distribution of distances, pointing to a range of protein conformations. All signals provide accurate distances, as illustrated in Figure 1 and in Figures S1–S5. The difference in appearance of the signal indicates that there is a distinct protein conformation in the post-hydrolysis intermediate in contrast to a wider distribution in the apo intermediate.

Average distances, reported in Table 1, reveal substantial reconfiguration in all three domains of MsbA. ATP hydrolysis fuels a closing motion in the intracellular domain of approximately 10–20 Å. The location of spin labels in three helices suggests that the distance changes reflect a concerted conformational rearrangement in the transmembrane segment. Site 103 in the intracellular part of helix 2 is particularly instructive, because the attached spin labels do not undergo changes in the motional state or in collision frequency with NiEDDA [29]. This indicates that there is no significant change in the local environment of the spin label, and the distance change at this site thus reflects a rigid body type movement of the backbone. An even larger distance change is reported at the NBD interface where spin labels at sites 539 move closer by almost 30 Å (we note that the spin label at site 539 is confined such that the uncertainty in distance is 0.3 Å). In contrast, an opening movement of 10 Å occurs between spin labels at site 61 in ECL1. Taken together, these distance changes suggest that alternating access of the chamber is induced by substantial movements on both sides of the transporter. We emphasize that the results for detergent micelles and liposomes given in Table 1 are sufficiently close to justify the use of (detergent) micelles in the study of MsbA. This was not at all obvious at the outset of the present study and highlights the advantage of pulse dipolar spectroscopy in allowing the measurements of distances in both environments.

**Table 1:**
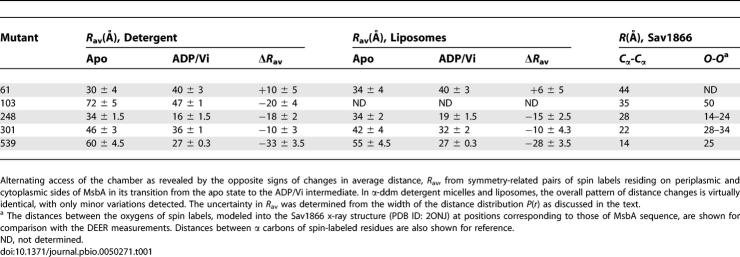
Amplitude of ATP-Induced Distance Changes in the Three Regions of MsbA

A similar pattern of distance changes in the three regions of MsbA is reported by fluorescein probes undergoing homotransfer [37,39] (Table 2). The probes were introduced in the same general locations as the spin labels, although the exact residues were adjusted to minimize perturbation by their larger molar volume, which affected reactivity and compromised stoichiometric labeling at sites such as 301. Unlike DEER, homotransfer is measured at ambient temperatures where the transporter samples all the conformers that are accessible. However, the widths of distance distributions in the apo state as well as for spin labels in the ECL1 loop support a significant range of conformations trapped in frozen samples. Comparison of Tables 1 and 2 shows a general agreement in the sign of the distance changes at both sides of MsbA. The absolute distances between fluorescein labels in liquid solution and spin labels in frozen media are in reasonable agreement to the extent imposed by the difference in the reporter groups, and the average nature of the distances calculated from steady-state fluorescence anisotropy. Zou et al. carried out a systematic comparison of distances calculated by the two methods and suggested that the primary factors accounting for the differences are the extension of the linking arm and the tendency of either probe to undergo specific interactions with neighboring main and side chains [39].

**Table 2:**
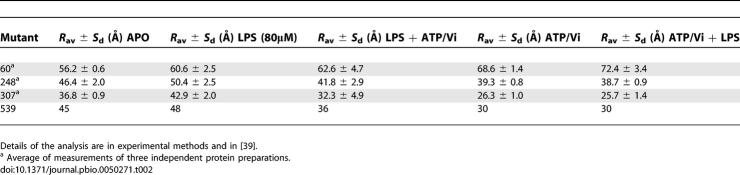
Distances between Fluorescein Labels in Various MsbA Intermediates

### Interpretation of the Distances in the Context of the Sav1866 Structure

**Figure 2:**
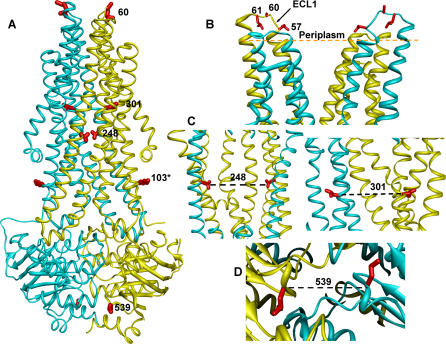
Sites of Spin Labeling in the Sav1866 Structure **(**A**)** Ribbon representation of Sav1866 structure highlighting sites of spin labeling using the numbering scheme of MsbA. (B) Locations of labeling sites 57, 60, and 61 in the ECL1 loop on the periplasmic side of Sav1866. (C) Relative orientations of sites 248 and 301 in a close up view through the closed chamber at the cytoplasmic side. The front helices are deleted to open the view of the two residues. (D) Top view of the NBDs in Sav1866 highlighting the location of 539.

We compared the distances measured in the ADP/Vi intermediate to the crystal structure of Sav1866. Figure 2 maps the sites of spin labeling onto the crystal structure of Sav1866 [13]. Distances were calculated by modeling the spin label side chain into the protein structure (e.g., Figure S6). At solvent-exposed sites, the spin label is not expected to have a preferred orientation relative to the backbone and can be represented as a cone projected along the C_α_-C_β_ bond with a 7-Å distance between the C_α_ and the nitroxide oxygen [43], although exclusions due to the tertiary contacts leading to “unusual” rotamers are possible [44,45]. In particular, at site 99, which is equivalent to 103 in MsbA, the spin labels point in opposite directions, and the predicted distance between the nitroxide oxygens is 50 Å, in close agreement with the experimental distance of 47 Å. A 47–53 Å distance range is obtained by modeling the g+ g+ g+ rotamer of the spin label visualized in a number of crystal structures [44].

At buried sites, repacking due to steric constraints between the main-chain and side-chain atoms biases the dihedral angles along the linking arm and results in a net orientation of the spin label that cannot be easily modeled. However, even in these cases, the deviations of the measured distance from the alpha carbon separation can be rationalized by the expected projection of the spin labels (Figure 2B). To illustrate this effect, we sampled the range of distances between spin labels at site 244 (248 in MsbA) by changing the torsion angles around the C_α_-C_β_ and C_β_-S_γ_ bonds. Whereas the sampling grid was selected to represent the extreme distances, it was not exhaustive and we did not attempt to assess the relative energies of spin labeled MsbA for every set of torsion angles. We found the modeled distance between the spin labels to vary from 14 to 24 Å (Table 1), which is shorter than the distance between the corresponding alpha carbons as expected from projecting the spin label along the C_α_-C_β_ vector (Figure 2B), but well in line with the experimental data in Figure 1B. At site 297 (301 in MsbA), the range of distances between the spin labels is 25 to 34 Å which is larger than the alpha carbon separation reflecting a relative outward projection of the spin labels (Figure 2B). Finally, we can model the spin labels at site 536 (539 in MsbA) in a conformation with no steric overlap to yield a separation of 25 Å that agrees well with the measured distance (Table 1).

### Effects of LPS on the Structure of MsbA

Similar to multidrug ABC transporters, a spectrum of potential MsbA substrates has been identified based on stimulation of ATP hydrolysis [46,47]. Among them is the substrate lipid A and a number of its processed derivatives including Ra LPS. The latter was used in the crystallization of MsbA [48] and was visualized bound to the external surface of the molecule. Ra LPS is relatively more soluble than lipid A, hence its use is more practical for spectroscopic analysis.

**Figure 3:**
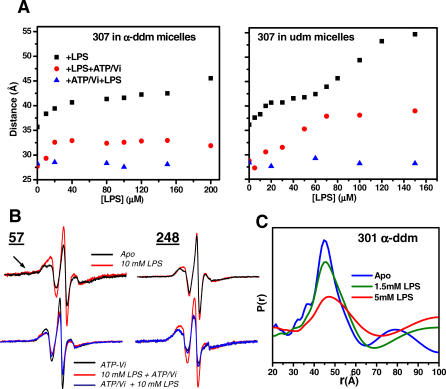
LPS-Induced Structural Changes in MsbA (A**)** Biphasic increase in distance between chamber-facing residues. The onset of the monotonic phase depends on the solubilizing detergent. Udm: undecyl-maltoside. **(**B**)** High concentration of LPS induces the appearance of a mobile component in the ESR spectral lineshape. **(**C**)** A population of transporters with an increased separation between monomers as reflected in the *P*(*r*) at site 301. This effect is inhibited by prior formation of the ADP/Vi intermediate. Subsequent ATP hydrolysis only partially restores the original lineshape. ESR samples were in 0.08% α-ddm.

LPS at a concentration used for stimulation of ATP hydrolysis [46] increases the distance between fluorescein labels at all sites, although the effect on the NBDs appears marginal (Table 2). The distance increase has the shape of an apparent binding isotherm in the lower range of LPS concentrations (Figure 3A). The effect is detergent-dependent: LPS titration in undecyl-maltoside, which has a higher critical micelle concentration (CMC) relative to α-ddm (dodecyl maltoside), shifts the curve to lower concentrations, and exposes an inflection point above which the distance increases monotonically (Figure 3A Figure S7). Given that LPS forms micelles at submicromolar concentrations, the inflection point may reflect a major structural transition as LPS progressively assumes the role of a solvent.

If LPS is acting as a substrate, then it is expected that ATP binding and/or hydrolysis will reduce its affinity [21,25]. Indeed, the LPS-induced distance increases are inhibited by prior formation of the ADP/Vi intermediate and the concomitant closure of the chamber (Figure 3A), which is consistent with a model where the LPS molecule or its head group interacts with residues at the cytoplasmic end of the chamber.

To further characterize the modes of LPS interaction with MsbA, we probed the structural changes induced by excess LPS concentration (1–10 mM) relative to the initial detergent. LPS induces the appearance of a mobile component in the ESR spectrum at all sites explored and leads to a new population of transporters with longer distances (Figure 3B). At the periplasmic site 57, the addition of LPS reduces the amplitude of dipolar splittings (arrow in Figure 3B), which reflects spin labels separated by less than 10 Å, implying an increase in distance.

An effective distance increase between residues 301 (Figure 3C) and 61 (Figure S8) for an LPS concentration of 1.5 mM (which is already in excess, as compared to a protein concentration of ∼100 μM) is also detected by DEER. The increase in distance at site 61 is more pronounced than for site 301, with virtually no change for site 248 (unpublished data). This may indicate preferential binding of LPS at the ECL1 loop region that is always accessible due to the spherical shape of micelles. In addition, the same sign of the distance change at sites 301 and 61 is not in line with the opposite signs of comparably large changes produced in opening or closing of MsbA by ATP hydrolysis, but is more indicative of a different structural change. Taken together, the data suggest that at these concentrations, LPS induces an increase in monomer separation in the transmembrane domain.

At an LPS concentration of 5 mM, there is no further increase in distances, but the pattern of DEER signals clearly changes and can be interpreted in two ways. The first, and most likely, explanation is that it reflects the presence of a second component with a much longer distance (>70 Å); this could imply the complete dissociation of the dimer. The second, less likely, explanation is that at excessive concentrations of LPS, lipids bridge two transporters at their periplasmic side (as was observed in the crystal structure of MsbA-ADP/Vi in presence of LPS in high concentration), leading to a more rapid decay of the DEER signal. But this case technically is more difficult to reconcile with the nearly uniform pattern of signal change for sites 61, 248, and 301 located at progressively larger distance from spin-labels residing on the suggested second dimer. A more detailed DEER study would be instrumental in establishing the precise nature of the mode of LPS interaction with MsbA

Hydrolysis of ATP subsequent to LPS addition resets the distance constraints close to those of the ADP/Vi as long as the LPS concentration is in the range of the binding isotherm (Figure 3A). The ensemble averaging of the homotransfer by steady-state anisotropy detection implies that the partial distance recovery may reflect a population of transporters that did not turn over ATP; i.e., have a separation similar to that obtained by LPS addition. This interpretation is reinforced by the multi-component nature of the ESR lineshape and its partial recovery at higher LPS concentrations. Notable is the decrease in the population of labels with dipolar coupling at sites 248 and 307 (unpublished data) if LPS is added before ATP and Vi are added (Figure 2C). In contrast, the ESR spectra of the preformed ADP/Vi intermediate are unchanged after the addition of up to 10 mM LPS, as illustrated in Figure 3B for site 248 (compare black and blue traces). Thus, high concentrations of LPS relative to the detergent result in substantial structural reorganization, which may reflect a solvent effect rather than the specific interaction of a substrate.

## Discussion

The nucleotide-bound structure of Sav1866 [13] has demonstrated that the post-hydrolysis conformation of ABC efflux transporters has an open chamber to the extracellular or periplasmic side [13]. What is less clear is the orientation of the chamber in the absence of nucleotides and whether the two NBDs undergo cycles of dimerization/dissociation [4,6]. Crystallographic analysis of isolated NBDs led to a model wherein ATP binding is required for NBD dimerization whereas its hydrolysis favors dissociation. The ATP-switch model proposes that the NBDs cycle between open and closed dimer conformations upon ATP binding although without dissociation or major reorientation relative to the transmembrane domain [27]. Crystallographic analysis of intact ABC importers has been particularly supportive of a limited separation between the NBD dimer during transport [14,15]. The structures suggest that the alternating access can be accommodated with little change in overall transporter architecture [14,15]. In this model, the two NBDs, which are independent subunits, are in contact throughout the transport cycle with relative movement confined to the P-loop and the signature motif. In the ADP-bound structure of Sav1866, the packing of the NBDs was interpreted as challenging dimerization/dissociation models [13].

**Figure 4:**
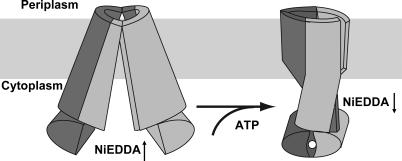
Schematic Illustration of the Conformational Changes Induced by the Formation of the High-Energy ADP/Vi Intermediate The cartoon recapitulates the domain swapping and twisting that characterizes the structure of Sav1866 (ADP/Vi intermediate) [13]. The apo intermediate has large separations between the two NBDs (Figure 1) which leads to high accessibility to the polar reagent NiEDDA on the cytoplasmic side as reported by Dong et al. [29]. Transition to the ADP/Vi intermediate reduces accessibilities and distances on this side of the transporter.

Our distance constraints obtained in detergent and liposomes provide a scale for the movements in MsbA that mediate the cycling of chamber accessibility reported by spin labels in the intracellular regions, ECL2, and along helices 2, 5, and 6 [29]. In conjunction with previously reported distance changes at site 57 [29], the data from sites 60 and 61 strongly indicate that the extracellular side of the transporter undergoes opening motion of large amplitude following ATP hydrolysis. This distance is well beyond the uncertainties imposed by possible label conformations. Spin labels at site 57 show distinct dipolar splitting in the continuous wave ESR [29] spectrum, implying closer proximity of ECL1 in the apo conformation relative to the nucleotide-bound structure of Sav1866. Similarly, the closing of the chamber in the cytoplasmic side that leads to reduction in NiEDDA accessibility upon ATP binding occurs through large movements, although in the opposite direction compared to the periplasmic side. While an outward/inward cycling of chamber accessibility can be accommodated in the constant contact model [15], the maximum predicted separation between the MsbA monomers is limited. The uniformly large magnitude of the distance changes reported here by two independent probes cannot be easily interpreted by this model. The variations in the amplitude of the distance changes between different sites in the cytoplasmic domain suggest that the relative movement is not a simple relative translation between the two MsbA monomers as noted by Dawson and Locher and schematically illustrated in Figure 4 [13].

More importantly, the distance change between the NBDs cannot be reconciled with constant contact models. The packing of the NBDs in the apo BtuCD structure represents their maximal separation during the cycle [14]. The structure predicts a 13-Å distance at the α carbons of 539, which is not consistent with our measured distance in apo MsbA. Similarly, comparison of the isolated NBDs of MalK in different nucleotide states reveals a limited association/dissociation cycle and predicts a distance change close to 4Å at the α carbon at the equivalent residue to 539 [6]. The scale of the experimentally measured distance change implies a significantly larger separation of the dissociated NBDs in MsbA as depicted in Figure 4.

It is possible that dissociation of the NBD dimer is a property of efflux ABC transporters reflecting a more substantial reconfiguration of the substrate chamber and the need to accommodate bulky substrates such as lipid A. In the Sav1866 structure, two helices in the ICD (IL) of one monomer contact the NBD of the opposite monomer, an interaction not observed in the BtuCD transporter. If these contacts were to be disrupted due to the opening in the ICD region as implied by our data (sites 103 and 248), this may destabilize the NBD dimer enough to allow its complete dissociation.

Our results provide direct structural insight into the interaction of LPS with MsbA. It is clear that the addition of LPS leads to structural rearrangements even at low concentrations. However, as with all lipophilic substrates, the results can be easily confounded by changes in the properties of the micelles and liposomes. In the case of MsbA, high concentrations of LPS induce pronounced structural rearrangements possibly reflecting a solvent-like effect. At all sites explored here, high concentrations of LPS increased mobility of spin labels. We are carrying out a systematic analysis of LPS effects on MsbA structure in detergents and liposomes (P Zou HS Mchaourab, unpublished observations).

When analyzed in the context of previous crystallographic and biochemical studies, our results are consistent with the initiation of the transport cycle by substrate binding to an open chamber at the cytoplasmic side of the transporter. After ATP binding, large-amplitude motion is required to form the ABC dimer, consistent with the two domains having significant conformational entropy in the apo intermediate and not being in contact throughout the cycle. In addition to the absolute distances reported in Table 1, this configuration is also supported by large NiEDDA accessibilities of residues on the cytoplasmic side in the apo intermediate [29]. Furthermore, the reanalyzed structure of apo MsbA shows a large open chamber and the two NBDs are separated by about 50 Å (G Chang, personal communication). Because the cellular concentration of ATP exceeds the *K*_m_ of MsbA, the open conformation of the apo intermediate is expected to be transiently populated. Current models of ATP hydrolysis propose that ATP turnover resets the conformation of the substrate binding site to high affinity [29]. Indeed, the accessibility profiles of spin labels in the ADP-bound and apo intermediates (J Dong and HS Mchaourab, unpublished results) are similar implying that the open state may be populated after ATP turnover and release of inorganic phosphate but before rebinding of ATP. In conjunction with previous accessibility data [29], our results support a model where a reversal of the chamber polarity gradient through an alternating access mechanism makes the two orientations of the substrate headgroup relative to the transporter energetically equivalent and initiates substrate translocation.

## Materials and Methods

### Purification and labeling of MsbA.

MsbA mutants were expressed and purified as previously described [29]. Briefly, Escherichia coli BL21(DE3) harboring the mutant plasmids were grown in minimal media and the protein expression was induced at 30°C. MsbA was extracted using α-ddm and purified by a two step nickel-affinity and size-exclusion chromatographies. The mutants were labeled with either the MTSSL (1-oxyl-2,2,5,5-tetramethylpyrrolinyl-3-methyl)-methanethiosulfonate spin label or MTS-fluorescein (2-[(5-fluoreceinyl) aminocarbonyl] ethyl methanethiosulfunate) (Toronto Research Chemicals; http://www.trc-canada.com/) [39]. Both fluorescein and spin-labeled mutants have retention times similar to the WT on the superdex 200 size-exclusion chromatography column. The spin-labeled mutants reported here were previously shown to turn over ATP at rates 20%–100% of the WT [29]. Reconstitution into liposomes was carried out as previously described [29] except that the lipid to protein molar ratio was adjusted to 2000/1.

For homotransfer, two samples were prepared for each mutant. Stoichiometrically labeled samples were prepared by addition of 10-fold molar excess of fluorescein twice over a period of 4–6 h. The reaction was allowed to proceed overnight at 4 °C. Underlabeled samples were prepared by adding 0.2 moles of fluorescein per mole of MsbA followed by addition of a 5-fold molar excess of a diamagnetic analog of the MTSSL (Toronto Research Chemical) to block the unreacted cysteines [49]. Labeling efficiencies were determined by comparing the absorbance at 280 nm to that at 492 nm. As shown previously, this ratio can be used to confirm labeling efficiency [39]. All stoichiometrically labeled samples had a 0.5 absorbance ratio.

Liposomes samples for spectroscopic analysis were in a 50 mM Hepes, 50 mM NaCl, pH 7.5 buffer. Ra LPS (Sigma-Aldrich; http://www.sigmaaldrich.com) was dissolved into the same buffer containing the appropriate amount of detergent. Typically, MsbA mutants were incubated with LPS at 37 °C for 15 min before or after formation of the ADP/Vi intermediate. The ADP/Vi intermediate was trapped by addition of 1 mM Vi following addition of ATP solutions containing 5 mM MgCl_2_ and incubated for 20 min at 37 °C.

### Distance measurement by pulse dipolar spectroscopy.

Distance measurements were carried out either on a home-built spectrometer at the National Biomedical Center for Advanced Electron Spin Resonance Technology (ACERT) facility at Cornell University, which operates at 17.3 GHz, or on a Bruker 580 pulsed ESR spectrometer, which operates at 9.36 GHz, using DEER with a standard four-pulse protocol [35] in both cases. For detergent samples, glycerol was added to yield 30% w/w prior to cooling. All experiments were carried out at 50–80 K. DEER signals were analyzed by the Tikhonov regularization and maximum entropy methods (MEM) [41,50] to determine average distances and distributions in distance, *P*(*r*), as illustrated in Figures S1–S5. The error in the distance was conservatively estimated by taking half of the *P*(*r*) width at 0.7 of the height.

### Distance measurement by fluorescence homotransfer.

Samples were analyzed on a steady state T-format fluorometer (Photon Technology International; http://www.pti-nj.com/). For each mutant, we collected steady-state anisotropy for a stoichiometrically labeled sample as well as an underlabeled sample. The latter serves as a reference wherein the steady-state anisotropy reflects the intrinsic reorientation of the probe. The extent of labeling was parametrized using the ratio of absorbance at 280 and 492 nm and compared to that expected based on labeling of T4L where the absolute extinction coefficient is available.

The fluorescence anisotropy, *r,* was measured by comparing the polarization of the emitted light to the polarization of the excitation light according to the equation:

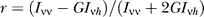
where *I*_vv_ and *I*_vh_ refer to the amplitude of fluorescence emission parallel and perpendicular to the plane of excitation light, respectively. The *G*-factor was determined for each sample to correct for bias in each channel.

Distances were calculated using an expression derived by Runnels and Scarlata [38,39]. We have calibrated this method using T4 lysozyme as a model protein system and demonstrated the correspondence of distances determined between spin labels and those determined by homotransfer [39].

## Supporting Information

Figure S1 Analysis of DEER Signals at Site 61. (A) Time domain DEER signals at site 61 in detergent and lipososmes. (B) Distance distributions calculated from the time domain data in (A).
(60 KB PPT)

Figure S2 Time Domain DEER Signals and Distance Distributions at Site 248 in Detergent Micelles (58 KB PPT)

Figure S3 Time Domain DEER Signals and Distance Distributions at Site 301 in Detergent Micelles and Liposomes (46 KB PPT)

Figure S4 Time Domain DEER Signals and Distance Distributions at Site 539 in Detergent Micelles (39 KB PPT)

Figure S5 Time Domain DEER Signals and Distance Distributions at Site 103 in Detergent Micelles (53 KB PPT)

Figure S6 Model of the Spin Label Side Chain at Site 99 of Sav1866 (1.5 MB PPT)

Figure S7 Effects of LPS on the Distance between Fluorescein Probes at Three Sites in MsbA (97 KB PPT)

Figure S8 Effects of LPS on the Distance between Spin Labels at Site 61 in Detergent Micelles (44 KB PPT)

## Abbreviations

ABC: ATP-binding cassette
ddm: dodecyl maltoside
DEER: double electron-electron resonance
ESR: electron spin resonance
LPS: lipopolysaccharide
NBD: nucleotide-binding domain
NiEDDA: nickel-ethylenediaminediacetic acid
P-gp: P-glycoprotein
Vi: vanadate
WT: wild type
